# Hepatic Inflammatory Pseudotumor—Focusing on Its Heterogeneity

**DOI:** 10.3390/diagnostics13172857

**Published:** 2023-09-04

**Authors:** Soo Ryang Kim, Soo Ki Kim, Yu-ichiro Koma, Motoko Sasaki, Akira Asai, Hiroki Nishikawa

**Affiliations:** 1 Department of Gastroenterology, Kobe Asahi Hospital, Kobe 653-0801, Japan; asahi-hp@arion.ocn.ne.jp; 2 Department of Pathology, Kobe University Graduate School of Medicine, Kobe 650-0017, Japan; 3 Department of Human Pathology, Kanazawa University Graduate School of Medicine, Kanazawa 920-8640, Japan; 4 The Second Department of Internal Medicine, Osaka Medical and Pharmaceutical University, Takatsuki 569-8686, Japan

**Keywords:** hepatic inflammatory pseudotumor, pathological findings, pathogenesis, differential diagnosis, heterogeneity

## Abstract

Hepatic inflammatory pseudotumors (IPTs) are defined as benign, non-malignant, non-metastasizing tumors characterized by the presence of myofibroblastic spindle cells, hetorogenous populations of inflammatory cells, particularly plasma cells, lymphocytes and macrophages, as well as locations of fibrosis and necrosis without cellular anaplasia or atypical mitoses. Despite subsequent reports in the references, hepatic IPT remains difficult to diagnose; while posing major issues specifically for its differential diagnosis compared with that of other various benign diseases and malignant hepatic tumors. Histopathological findings are always a requisite for confirming the diagnosis, particularly given that the pathogenesis of IPT remains ambiguous to date. Hepatic IPT is a heterogeneous entity in terms of its clinical features, pathological findings, and pathogenesis. Once the diagnosis is confirmed, however, needless surgery such as wedge resection and lobectomy should be avoided. Here, we discuss the heterogeneity of hepatic IPT, its clinical features, pathological findings, and pathogenesis, and describe its differential diagnosis.

## 1. Introduction

Inflammatory pseudotumors (IPTs) are observed mostly in the lung, and rarely in outside pulmonary organs such as the liver [1,2]. In consecutive cases of 84 outside pulmonary IPTs, only 7 (8%) were located in the liver [3]. Histological analysis shows that IPTs are characterized by the infiltration of fibroblasts or myofibroblasts and inflammatory cell infiltration, regardless of their tissues of origin [1,2,3,4,5]. The inflammatory cells comprise mainly polyclonal lymphocytes and plasma cells [6]. Hepatic IPT is defined by benign, non-malignant, non-metastasizing nodules characterized by the existence of myofibroblastic spindle cells, heterogeneous populations of inflammatory cells, specifically plasma cells, lymphocytes and macrophages, as well as areas of fibrosis and necrosis without cellular anaplasia or atypical mitoses [7,8,9]. Hepatic IPT was first described in 1953 [10]. Irrespective of many following papers in the references [11,12,13,14], hepatic IPT remains difficult to diagnose, while posing major issues, specifically for its differential diagnosis compared with that of other benign diseases [15]. However, no genetic mutations have been reported for differential diagnostics between IPT and other diseases so far.

Although histological examinations are a requisite for confirming the diagnosis, the challenge in diagnosing IPT is amplified in that most reported cases have been diagnosed by surgical procedures such as wedge resection and lobectomy [15]. Moreover, since neither imaging modalities nor biochemical tests provide conclusive evidence of IPT [15], it is not easy to distinguish IPT from malignant liver tumors including hepatocellular carcinoma, cholangiocellular carcinoma, cholangiolocellular carcinoma and metastatic liver cancer. Indeed, liver tumor markers such as AFP, PIVKA II, and CEA were almost within normal limits in the reported references [16,17]; however, liver function tests such as ALT and AST showed multifield increases due to inflammation in the reported references [17].

The etiology of IPT remains to be clarified to date, especially in the absence of a written basis of its having relations with any specific disease different from phlebitis [2] and Crohn’s disease [18], as demonstrated in some papers. Infection by microorganisms, such as bacteria and viruses, and autoimmune reactions have been indicated in the clinical course of some IPTs, although their etiology has not been well documented [19,20]. Hepatic IPT is a heterogeneous entity in terms of its clinical features, pathological findings and pathogenesis.

This review focuses on hepatic IPT and discusses its heterogeneity, clinical features, pathological findings and pathogenesis, and describes its differential diagnosis.

## 2. Pathological Findings and Clinical Features

**Figure 1:**
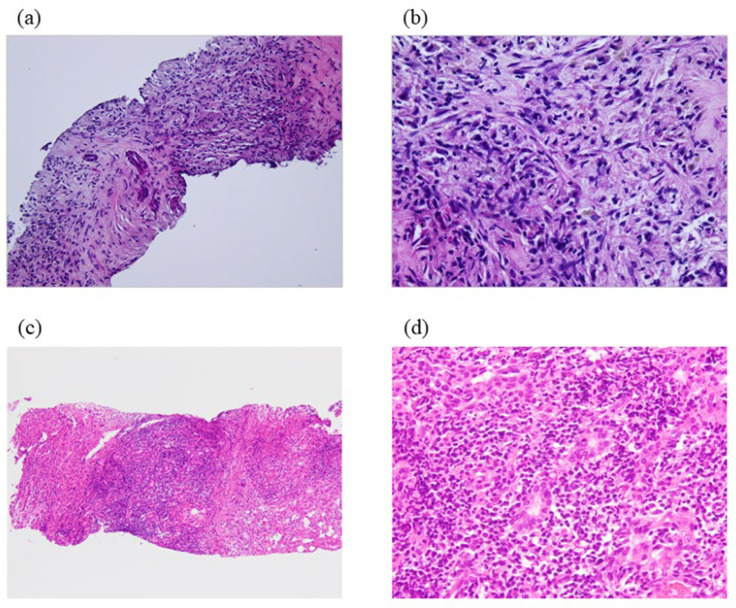
Histopathology of hepatic IPT, fibrohistiocytic type. (**a**) Low power × 100 HE; (**b**) high power × 400 HE. Proliferation of fibroblasts, lymphocytic cells, plasma cells, and foamy macrophages on the background of collagen fibers are observed. Histopathology of hepatic IPT, lymphoplasmacytic type. (**c**) Low power × 100 HE; (**d**) high power × 400 HE. Diffuse lymphoplasmacytic infiltration is observed.

Through pathological analysis, IPT has been classified into two types: fibrohistiocytic (Figure 1a,b) and lymphoplasmacytic (Figure 1c,d). Fibrohistiocytic IPTs are featured by xanthogranuloma, multinucleated giant cells, and neutrophilic inflammation, observed most commonly in the peripheral hepatic parenchyma as tumor-forming nodules [6]. According to previous papers on hepatic IPTs, the lymphoplasmacytic type appears to refer to what we call plasma cell granulation tissue [4,21]. Additionally, it was shown that the two types of hepatic IPT had other clinical and pathological characteristics, such as immunohistochemical findings, clinical features, and the place of the nodules, implying the correctness of this classification theory.

The etiology of fibrohistiocytic-type hepatic IPT continues to be only hypothetical. This group demonstrated xanthogranuloma accompanied with many extraordinarily large nucleated cells and neutrophils. Of interest, there were many αSMA-positive myofibroblastic cells inside the nodule, indicating a progressing fibrous course.

Inside the nodule, bile ducts were damaged and venous divisions were closed, indicating a damaging inflammatory course in the etiology of this group of liver IPT.

Cholangitis associated with periductal inflammation near tumors of IPT may be next to the inflammatory course. This group could be the last version of various and damaging inflammation courses in the liver, and various pathogeneses may be associated with this liver IPT. Of interests, two of the fibrohistiocytic nodules had many IgG4-positive plasma cells, though the other histopathological characteristics of these two nodules were truly different from those of IgG4-related disease. It is supposed that the two cases were histopathologically different from the IgG4-related disease, though it might be plausible that they initially contained IgG4-related lymphoplasmacytic IPT or sclerosing cholangitis, and secondary variations including leakage of bile developed.

Fibrohistiocytic IPT has been identified in 30 patients, and it has been reported that the inflammatory infiltrates were dominated by lymphocytes and plasma cells, and by prominent histiocytic infiltrates in 22 of 30 cases. Neutrophils have been found in 12 patients, with microabscesses observed in 8, and storiform pattern of fibrosis has been observed in 14/30 cases; although obliterative phlebitis was not identified, microorganisms were observed in biopsy tissue cultures in 4 of 9 cases [22].

Contrastingly, lymphoplasmacytic IPTs demonstrate broad lymphoplasmacytic and characteristic eosinophilic infiltration, and are all shown near the liver hilum. Interestingly enough, IgG4-positive plasma cells were demonstrated in a broad manner in every lymphoplasmacytic case. Lately, much focus has been directed towards IgG4-related disease, which is a disease classification first suggested in terms of autoimmune pancreatitis [23,24,25]. IgG4-associated morbidity can exist in many tissues including the salivary tissue (chronic sclerosing sialadenitis) [26], lung (IPT or interstitial pneumonitis) [27,28], and retroperitoneum (retroperitoneal fibrosis) [29,30]. It has pathohistological features such as lymphoplasmacytic inflammation, uneven fibrosis, eosinophilic inflammation, and enriched IgG4-positive plasma cells. Lymphoplasmacytic IPTs had all of these pathohistological characteristics of IgG4-related disease. Moreover, venous closure with little infiltration and cholangitis with no periductal fibrosis are often seen in the fibrohistiocytic type, although occluded phlebitis and cholangitis with periductal fibrosis are usual characteristics of the lymphoplasmacytic type. Interestingly enough, IgG4-positive plasma cells are much more common in the lymphoplasmacytic than in the fibrohistiocytic type. The lymphoplasmacytic type is specific, and may be classified as a so-called IgG4-related diseases. Contrastively, the fibrohistiocytic type could still refer to a diverse group of diseases, and may appear histopathologically different from IgG4-related tumors, though cases with enriched IgG4-positive plasma cells need to be discriminated from IgG4-related disease [6].

Clinical features of hepatic IPT differ in terms of average age, man/woman ratio, tumor size, and tumor location of each type of IPT. The fibrohistiocytic type occurs at the same rate in both sexes, although many patients of the lymphoplasmacytic group are male. The place of the nodule differs in the two groups. The lymphoplasmacytic group is more usually seen in the left lobe. Eight nodules (80%) of the fibrohistiocytic group are placed in the hepatic parenchyma, demonstrating nodule-forming features. Contrastingly, every IPT of the lymphoplasmacytic type is observed in the liver hilum, and extends along hilar bile ducts [6]. Macroscopically, the fibrohistiocytic IPT resembles nodule-forming intrahepatic cholangiocellular carcinoma, although the lymphoplasmacytic IPT shows hilar nodules that are almost the same as those in periductal infiltrating-type hilar in cholangiocellular carcinoma. Interestingly, the clinical condition also differs between the two types. In total, 80% of cases with fibrohistiocytic IPT have shown non-objective complaints, including high temperature, abdominal pain, and general fatigue, although 83% with lymphoplasmacytic IPT have incidentally been found, through routine laboratory testing, to exhibit abnormal liver function (Table 1) [6].

## 3. Differential Diagnosis

### 3.1. Hepatic Inflammatory Myofibroblastic Tumor (IMT)

Hepatic IMT is a rare pseudosarcomatous infiltration of spindled myofibroblast cells mixed with changeable numbers of inflammation cells. From the first discovery in the lung, IMT has been found at multitude places outside of the pulmonary region, most remarkably inthe mesentery of the small bowel and the omentum of children and younger people [31].

The WHO defines IMT as a clear fibroblastic/myofibroblastic neoplasm of medium biological capability with remarkable amount of inflammatory infiltrates, mainly lymphocytes and plasma cells [32]. According to the WHO’s description of IMT, localization is described as follows: The small bowel and colon are the most routinely cited gastrointestinal locations, followed by the stomach. The esophagus, pancreas (direct extension from retroperitoneum), appendix, and liver are rare locations. The submucosa, muscularis propria, or mesentery may be affected [33]. Epithelioid inflammatory myofibroblastic sarcoma has a remarkable tendency towards the mesentery of the small bowel and omentum [34]. Additionally, some IMT cases of biliary tract origin are reported [35,36,37,38,39,40,41,42,43,44,45,46,47,48,49,50,51,52].

Previously, hepatic IMT had been regarded as a synonym for IPT, but is currently considered merely one type of hepatic IPT [53]. An IMT includes another classification that may demonstrate overlapping characteristics with fibrohistiocytic IPT [22].

After neglecting malignant disease, Arora et al. specified 30 cases with the fibrohistiocytic variant of hepatic pseudotumor (FHVHPT) from three institutions between 2009 and 2019. They analyzed clinicopathological findings, hepatic function examinations as well as culture consequences and radiological data. Hematoxylin and eosin-stained specimens were assessed for type of inflammation as well as its cellular component. Immunohistochemical studies for IgG4 and IgG were performed in all subjects. The average age of the 30 nodules featured as FHVHPT was 56 years (range: 23 to 79 years). In total, 19 patients demonstrated a single nodule; 11 were plural. The mean largeness of the nodule was 3.8 cm (range: 1 to 7.5 cm). In radiological imaging studies, a malignant course or metastasis was the chief diagnostic priority (*n* = 15, 50%). The most usual complaint was abdominal pain (*n* = 14/30); 8 patients showed complaints coincident with an infectious process, such as high temperature. The inflammatory infiltration was occupied by lymphocytes and plasmacytes, and in the majority of patients, a remarkable histiocytic inflammation was seen (22/30). Neutrophils were shown in 12 patients, with microabscesses shown in 8. A storiform sample of fibrosis was observed in 14/30 patients; occluded phlebitis was not clarified. Culture clarified a bacterium in four of the nine patients assessed. The average IgG4 count was 9.3 per high-power field (HPF) (range: 0 to 51), with 9 of the 26 (35%) biopsies demonstrating >10 IgG4 positive plasmacytes per HPF. The average IgG4 to IgG proportion was 8% (range: 8% to 46%). A hepatic resection was done in four subjects. On wide-range antibiotics (*n* = 14) the nodules either disintegrated or diminished in largeness. Eight subjects did not undergo specific treatment; however, the nodule(s) diminished naturally in six subjects, and showed no change or diminished in size in two subjects. Remarkably, no cases demonstrated evidence of a liver recurrence. FHVHPT, a tumefactive nodule that simulates liver neoplasia, is histopathologically characterized by a fibrohistiocytic infiltrate. Most cases of FHVHPT show the organizing process of liver abscess, and can be reasonably treated with antibiotic treatment [22]. Though many inflammatory myofibroblastic nodules demonstrate fewer inflammatory cells, the scarce presence of histiocytes, and atypia in the spindle cell element, a few of those nodules may have less clear atypia, and are instead occupied by a lymphoplasmacytic inflammatory infiltration.

Chougule et al. focused on the histopathologic overlap between the two diseases, and focused on the significance of a fusion examination in the discrimination of the IgG4-related nodule from IMT. All seven IMTs demonstrated a thick lymphoplasmacytic infiltration and storiform-pattern fibrosis, with occluded phlebitis reported in three patients. The neoplastic stromal cells comprised <5% of overall cellularity, and stromal atypia was either absent or partial and slight. Increased counts of IgG4-positive cells and increased IgG4 to IgG proportions were clarified in every case. Four patients demonstrated ALK associated dysfunctions, while two patients demonstrated ROS1 and NTRK3 fusions. One nodule was negative for known IMT-related gene fusions. All 56 IgG4-related disease subjects were negative for ALK and ROS1 on immunohistochemical analysis; 6 subjects were negative on the fusion assay.

Highly inflammatory IMTs are not distinguishable from IgG4-related disease either histologically or on the immunohistochemical findings for IgG4. This demands the close examining of patients with no evidence of single-organ IgG4-related disease for IMT, and the diagnostic algorithm should contain ALK and ROS1 immunohistochemical finding and, in chosen subjects, an NGS-based fusion examination that includes known IMT- related gene fusions [54].

The judicious use of immunohistochemical findings for ALK, ROS1, and NTRK may aid in the diagnosis of IMT [22,54,55]. However, it should be considered that immunohistochemical stains for ROS1 and NTRK may not offer strong substitute signs of IMT; assays that discover gene fusions would meet the criteria the best. It should also be acknowledged that gene fusions are discovered in about 50% of IMTs in adults, strengthening the need to cautiously evaluate atypia inside stromal cell division [22,54].

Owing to the eminent inflammatory infiltrates and related general symptoms in the minority of patients with hepatic IMT, a viral etiology has been suggested, but the evidence in this regard is not convincing [56,57,58]. Indeed, hepatic IMT may occur after trauma, operation, or infection, confirming the reactive process of the nodule [31,59].

Histopathologically, the spindled myofibroblast and inflammatory cells show three histopathologic groups in variable ratios in liver IMT [31].

Firstly, the myxoid vascular pattern comprises roughly placed, stellate-to-plump spindle cells in an edematous, myxoid bases with an uneven network of tiny blood vessels and inflammatory cells, similar to nodular fasciitis or granuloma.

Secondly, the compact spindle cell pattern is characterized by a compact interlacing fascicular or storiform spindle cell infiltration with some extent myxoid and collagenized places, combined with an inflammatory infiltration composed mostly of plasma cells. This pattern was compared to fibrous histiocytoma and smooth muscle malignancy; collections of foamy histiocytes may also be present.

Thirdly, the hypocellular fibrous sample is characterized by platelike collagen, less cellularity, and scanty inflammation with lymphocytes and plasmacytes cells caught in a thick eosinophilic matrix. This pattern is thought to simulate a scar or a desmoid-type fibromatosis. Rough psammomatous calcification or osseous metaplasia can be discovered inside the fibrous places. In most formative patterns, the spindle cells do not demonstrate nuclear atypia [31].

The cytoplasmic ALK response is restricted to the spindle cell element of the nodule and can be associated fairly well with the ALK rearrangements detected by fluorescent in situ hybridization [60]. p53 immunopositivity is seldom seen, but has been reported in relation to recurrence and malignant transformation [60]. Although hepatic IMTs of the liver may simulate malignant tumors from the clinical and radiological view, they commonly do not recur after full surgical operation. In less than 60% of cases, hepatic IMTs of the liver are related to a gene rearrangement containing the ALK gene at chromosomal locus 2p23 [31]. Nonetheless, few subjects with local recurrence and an active clinical course have been described, especially in the small subset of subjects in whom full resection is unfeasible, and regression or response to prednisolone and nonprednisolone anti-inflammatory medicines have been reported (Table 2) [31].

### 3.2. Primary Sclerosing Cholangitis (PSC)

A fibrous-obliterative bile duct lesion characterized by an “onionskin” pattern of periductal fibrosis near middle-sized or bigger bile ducts has been described, associated with degeneration and atrophy of the epithelial interior and the ultimate restoring of the bile duct by fibrous cords [61]. Lymphoid follicles or their aggregates may be present, but granulomas are rarely observed. Tiny bile ducts may demonstrate degenerative epithelial changes or may be surrounded by a ring of edematous or hyaline fibrosis [61]. The classic onionskin fibrosis is seen in less than 10% of biopsy specimens from cases with PSC, but when observed, it is nearly pathognomonic [62]. Compared with the lymphoplasmacytic type of IPT, inflammation is severe and conspicuous on the inner side of the bile duct and pseudotumor due to the amelioration of inflammation that rarely occurs in the xanthogranulomatous type of PSC (Table 2) [6].

### 3.3. Follicular Dendritic Cell Tumor (FDC)

FDC tumors are markedly female-predominant, rare tumors that can contain lymph nodes or extranodal sites [63] and show a wide range of histopathologic features and activity; however, the intraabdominal ones commonly have an unfavorable outcome [63]. The cases comprised 10 females and 1 male (age range, 19–61 years; average, 40 years) who presented with abdominal fullness or pain. Six cases had general complaints including remarkable weight decrease, high temperature, or fatigue. All nodules were observed in the intra-abdomen space: liver (*n* = 7), spleen (*n* = 3), and peripancreatic place (*n* = 1). Among the nine cases with follow-up, six cases survived and got well, one case suffered recurrence after 9 years, and two cases had repetitive recurrence for many years [63]. Macroscopically, the nodules are commonly singular and fleshy, interrupted by places of bleeding and necrosis. Histological examination showed the tumors dispersed in a background of abundant lymphocytes and plasma, spindle or ovoid cells associated with vesicular nuclei and clear nucleoli. The extent of nuclear atypia changed, and some nuclei appear deformed or similar to Reed–Sternberg cells. Locally, the cell fascicles are spindle-shaped. The atypical cells are immunopositive for FDC signs including CD21/CD35, CD23, and CNA.42. The in situ hybridization of the Epstein–Barr virus (EBV)-encoded RNA has been positive in every case, mainly focusing on = spindle cells and their atypia. EBV-latent membrane protein-1 is commonly observed, albeit frequent partially and slightly (Table 2) [63].

### 3.4. Hepatic Pseudolymphoma

Distinguishing hepatic IPT from hepatic pseudolymphoma is challenging [64,65,66]. A case of pseudolymphoma (reactive lymphoid hyperplasia) of the liver in a 72-year-old man with chronic hepatitis C has been reported. Radiological findings, including ultrasound, CT, dynamic CT, and MRI, indicated liver cancer. A nodule-like lesion was therefore operated. Macroscopically, the nodule was to some extent poorly defined and sized 15 mm × 17 mm [65]. Another case of pseudolymphoma (reactive lymphoid hyperplasia) of the liver in a 66-year-old woman was reported. A nodule-like tumor was accidentally found in the liver during clinical observation of diabetes mellitus. The liver nodule was operated because malignant lymphoma was suggested after a needle biopsy. Macroscopically, the nodule was well-defined and sized 1.0 cm × 1.5 cm × 1.0 cm [66]; however, the latter is pathologically totally different from the former. Histopathological examination shows hepatic pseudolymphoma characterized by the existence of hyperplastic lymphoid follicles with polymorphic and polyclonal cell sets composed of tiny full-grown plasma cells, histiocytes and stromal fibrosis [65]. Immunohistological examination has revealed that the lymphocytes composed of many germinal centers are chiefly composed of L-26-positive B cell lymphocytes. The lymphocytes circumscribing the germinal centers are chiefly UCHL-1-positive T lymphocytes. The B cells in the lymphoid follicles stain positive for both κ and λ light chains at sequential recurrences, implying polyclonal and non-malignant characteristics (Table 2) [65,66].

## 4. Pathogenesis

(1) Although the pathogenesis of IPT is still unclear, several hypotheses have been proposed.

The relation between IPT and bacterial infection:

Associated conditions have been observed in 22 of 31 IPT cases at the Armed Forces Institute of Pathology, and a liver abscess or its remnants in 7 cases [67]. Cultures from 4 of these 22 lesions yielded various bacteria (*Escherichia coli*, *Klebsiella*, *Bacteroides*, *Proteus*, and unidentified anaerobes). *Klebsiella pneumoniae* have been isolated from one case [68] and *Bacteroides corrodens* from another [4]. Cultures of liver biopsy tissue of nine cases showed no growth in five cases, but one of the following organisms was identified in each of the other four cases: *Streptococcus anginosus*, *Klebsiella*, *Enterobacter*, and *Staphylococcus intermedius*. No parasitic infection was observed. Histological analysis, however, disclosed significant differences between positive and negative culture results. Most patients responded to antibiotics, although several displayed spontaneous resolution of the lesion(s) [22].

(2) The relation between IPT and post-infection and inflammatory processes:

A clinicopathological study of nine cases with IPT of the liver was written. The ages of the cases ranged from 22 to 83 years old, with a man to woman proportion of 8 to 1. Their complaints were of intermittent high temperature and abdominal pain, and examination results on admission indicated an inflammatory course. The single or plural well-defined nodules were shown by lately advanced radiological examinations. Hepatic resection, laparoscopic resection, needle biopsy, or autopsy was done in all nine cases under the diagnoses of hepatic cancer, metastatic hepatic cancer, or hepatic abscess. Histopathologically, these nodules were composed of thick hyalinized fibrosis and/or infiltration cells composed of many foamy histiocytes, lymphocytes, and plasma cells. Occluded phlebitis of relatively big divisions of the portal vein was discovered, thus providing a diagnostic aid to differentiate from primary liver tumors in radiological studies [2].

Clinicopathological analyses of nine patients with hepatic IPT revealed that two cases died of causes speculatively associated with this disease, implying poor prognosis in some cases, contrasting with the commonly good outcomes seen in the past literature [2]. Considering the clinicopathological features of IPT and the histories of four patients who had been in Southeast Asian countries or India, infection could have been induced by microorganisms passing into the portal vein of the liver.

Also described is a patient with hepatitis C virus (HCV)-related IPT with high temperature, leukocytosis and medium positive C-reactive protein indicating inflammation, 1 year before the discovery of a low echoic hepatic IPT [64]. A report of hepatic IPT in a 75-year-old woman with chronic hepatitis C whose radiological findings were similar to hepatic cancer has been given. A US-guided needle biopsy showed a chiefly wide and polyclonal infiltration of lymphocytes positive for common leukocyte antigens (Panlymphocyte cells), L-26 (B celllymphocytes), and UCHL-1 (T cell lymphocytes), negative for both κ and λ light chains and fewer infiltrated neutrophils and histiocytes. No lymphoid follicles were discovered [64].

(3) The relation between IPT and eosinophilia:

In an IPT case, laboratory tests have shown conspicuous, marked peripheral eosinophilia [15], indicating hypereosinophilic syndrome (HES), which is diagnosed when the relevant data are in the following range: constant eosinophilia (1500 eosinophils/mm^3^) for at least six months before or at dying with signs and symptoms of HES disease; excluding of other known causes of eosinophilia; organ system involvement or abnormality due to eosinophil inflammation or otherwise unexplained [69].

Sasahira et al. reported an IPT of the liver, wherein a 59-year-old man was admitted with obstructive bilirubinemia, remarkable eosinophilia (1343 /mm^3^) and hypergammaglobu-linemia (4145 mg/dL). Radiological examination showed enlargement of the intrahepatic bile duct, narrowing of the common bile duct with widely spread wall thickening, gallbladder wall thickening, uneven stenosis of the pancreatic duct, and enlargement of the pancreatic tissue. Many hepatic nodules were also found and identified as IPT with biopsied tissues. Six months after, the unusual characteristics of the biliary tree were significantly ameliorated by the oral intake of steroids, and the hepatic nodules disappeared. The enlargement of the pancreatic head was also ameliorated. The peripheral eosinophil number stabilized within normal limits. IPT related to autoimmune pancreatitis (AIP) has not been reported in the references. The clinical characteristics of the reported patients are the same as those of pancreatic tumor with hepatic metastasis [15]. In the reported case of peripheral eosinophilia, an eosinophils count lower than 1500/mm^3^ persisted for only 2 months, despite ruling out parasitic infection as a possible cause of eosinophilia. Nevertheless, eosinophilia might play a role in the etiology of the clinical characteristics of IPT [15].

(4) The relation between IPT and hepatitis B virus (HBV):

The analysis of a post-surgery retrospective study of 114 patients with hepatic IPT, recorded from July 2006 to July 2012 and comprising 69 men and 45 women (mean age 53.14 ± 10.98 years) with normal laboratory values, revealed 16 hepatitis B surface antigen (HBsAg)-positive patients and 8 with HBV-related liver cirrhosis. The data were statistically analyzed using the Chi-square test [8]. Most reported complaints were abdominal pain (59/144, 41.0%), high temperature (48/114, 42.1%), abdominal fullness (35/144, 24.3%), and weight decrease (12/144, 8.3%). The laboratory data were within normal limits. In total, 16 patients were HBsAg plus and 8 suffered from liver cirrhosis. The majority of the nodules were placed in the right lobe (79/114, 69.3%), 33 in the left lobe, and 2 in the caudal lobe. IPT diagnoses are difficult, and non-aggressive therapy should be prioritized when the diagnosis is defined. Computed tomography (CT) and magnetic resonance imaging (MRI) appear to have almost the same diagnostic ability [8]. The relation between IPT and HCV has, however, not been reported except by Kim et al. [64].

(5) The close relation between IPT and IgG4-related immune reactions:

Serum IgG4 values are elevated specifically in cases with AIP compared with those harboring other inflammatory or neoplastic diseases of the pancreatobiliary organ [23]. AIP is thought to show nodular enlargement of the pancreatic tissue, and was considered as pancreatic IPT [70]. Pathological studies have demonstrated that AIP is a lymphoplasmacytic sclerosing pancreatitis, characterized by significant lymphoplasmacytic inflammation, uneven fibrosis, occluded phlebitis, acinar atrophy, a relation to sclerosing cholangitis, and the degree of inflammatory course to peripancreatic fatty tissue [71,72]. Many IgG4-positive plasmacytes are easily discovered in inflamed places by immunohistochemistry, implying that IgG4 is not only a serum sign, but also one of the etiologic causes of AIP [30,73].

Hepatic IPT associated with sclerosing cholangitis has exactly the same pathological characteristics as those of AIP [74]. Cases have shown aggressive lymphoplasmacytic inflammation in many IgG4-positive plasma cells, peripheral phlebitis, and irregular fibrosis. The reporters have supposed that specific immunological systems associated with IgG4 cause nodular inflammation in the hepatic and pancreatobiliary organs [74]. Interestingly enough, lung and breast IPTs have the same histopathological characteristics, such as numerous IgG4-positive plasma cells [27,75]. The foregoing reports have speculated a histopathological and immunologic similarity between IgG4-related disease and IPT. Unclear, however, is whether all hepatic IPTs are associated with IgG4, or whether they can be histopathologically identified on the basis of IgG4-positive cells.

## 5. Conclusions

As described above, hepatic IPT is a heterogenous clinical entity in terms of clinical features, pathological findings and pathogenesis. Several diseases, including benign and malignant ones, require differential diagnosis. The outcome of this nodule is thought to be good in most cases. Today, a number of therapy choices such as antibiotics [76], nonsteroidal anti-inflammatory therapies and steroids have been described for hepatic IPT [77]; however, the natural disappearance of the tumor after several months has been presented [78,79]. Several liver tumor cases have been operated on or transplanted [80,81,82], and some could have been spared operation if diagnosis before operation had been implemented.

Once the diagnosis is determined, nonessential surgery such as wedge resection and lobectomy should be avoided. Hepatologists engaged in the field of liver tumors should be vigilant with regard to hepatic IPT.

## Figures and Tables

**Table 1:** Two types of inflammatory pseudotumor (IPT).

	Pathological Findings	Clinical Features
Fibrohistiocytic	Dominant lymphocyte and plasma cell infiltration and prominent histiocytic infiltration Xanthogranulomatous inflammation, multinucleated giant cells, and neutrophilic infiltration with microabscess Storiform pattern of fibrosis Frequent venous closure inside less inflammation and cholangitis with no periductal fibrosis	Gender: both genders involvedLocation and gross findings: mostly occurred in the peripheral hepatic parenchyma as nodule-forming lesions similar to intrahepatic cholangiocellular carcinoma Symptoms and examination: fever, abdominal pain, and general malaise
Lymphoplasmacytic	Widely spread lymphoplasmacytic infiltration and conspicuous eosinophilic infiltration seen near the hepatic hilum Obliterative phlebitis and cholangitis accompanied by periductal fibrosis Numerous IgG4-positive plasma cells	Gender: many cases involved menLocation and gross findings: more commonly observed in the left lobeSymptoms and examination: dysfunction by routine laboratory testing

**Table 2:** Differential diagnosis of hepatic inflammatory pseudotumor.

Diagnostics	Characteristics
Hepatic inflammatory myofibroblastic tumor	The three histologic patterns are listed below.The first is the myxoid-vascular pattern.Characteristics: Resembling nodular fasciitis or granulation tissue; Composed of loosely prepared, stellate-to-plump spindle cells inside an edematous, myxoid background with an uneven netting of small blood vessels and inflammatory cells. The second is the compact spindle cell group.Characteristics: A closely interlacing fascicular or storiform spindle cell proliferation inside variable myxoid and col lagenized areas; Associated with an inflammatory infiltrate consisting mostly of plasmacytes. The third is the hypocellular fibrous group.Characteristics: Platelike collagen, less cellularity, and to some extent weak inflammation within lymphocytes and plasmacytes within a dense eosinophilic matrix. Immunohistochemistry for ALK, ROS1, and NTRK may aid in the diagnosis of IMT.
Primary sclerosing cholangitis	The fibrous-obliterative pattern is characterized by an “onionskin” pattern of periductal fibrosis near medium-sized or bigger bile ducts, associated with degeneration and atrophy of the epithelial interior and accidental replacement of the bile duct by fibrous cords.Lymphoid follicles or aggregates may be present, but granulomas are rarely detected. Small bile ducts may show degenerative epithelial changes or may be surrounded by a ring of edematous or hyaline fibrosis.
Follicular dendritic cell tumor	Tumors are dispersed in a background of abundant lymphocytes and plasma, spindle or ovoid cells with vesicular nuclei and distinct nucleoli. The extent of nuclear atypia varies, and some nuclei appear deformed or simulate Reed-Sternberg cells. The atypical cells are immunoreactive to FDC signs such as CD21/CD35, CD23, and CNA.42. The Epstein–Barr virus (EBV)-encoded RNA is positive in every case of in situ hybridization.
Pseudolymphoma	The hyperplastic lymphoid follicles with polymorphic and polyclonal cell populations consisted of small mature plasmacytes, histiocytes and stromal fibrosis.Lymphocytes consisting of many germinal centers are chiefly composed of L-26-positive B cell lymphocytes. The lymphocytes encircling the germinal centers are chiefly UCHL-1-positive T lymphocytes. The B cells in the lymphoid follicles are positive for both κ and λ light chains at sequential frequencies.

## Data Availability

Not applicable.

## Abbreviations

IPT: inflammatory pseudotumors; HES: hypereosinophilic syndrome; AIP: autoimmune pancreatitis; FHVHPT: fibrohistiocytic variant of hepatic pseudotumor; IMT: inflammatory myofibroblastic tumor; ALK: anaplastic lymphoma kinase; ROS1: proto-oncogene tyrosine-protein kinase; NTRK: neurotrophic tyrosine receptor kinase; NGS: next-generation sequencing; PSC: Primary sclerosing cholangitis; FDC: follicular dendritic cell tumor.
